# Did Material Properties Contribute to the 1284 Collapse of Beauvais Cathedral? A Comparative Assessment of Stone Masonry, Mortar Behaviour and Structural Vulnerability

**DOI:** 10.3390/ma19214659

**Published:** 2026-11-01

**Authors:** Rubén Rodríguez Elizalde

**Affiliations:** Higher Polytechnic School of Technology and Science, Camilo José Cela University, C/Castillo de Alarcón, 49, Urbanización Villafranca del Castillo, Villanueva de la Cañada, 28692 Madrid, Spain; ruben.rodriguez1@ucjc.edu

**Keywords:** Beauvais Cathedral, chalk, lime mortar, gothic masonry, structural collapse, creep, differential shortening, Monte Carlo analysis, reliability index, conservation

## Abstract

The partial collapse of the choir vaults of Beauvais Cathedral in 1284 has been explained through structural and constructional hypotheses, but the role of the materials has never been tested against laboratory data. The objective of this study is to quantify, with a transparent probabilistic framework, whether the properties of the stone and the lime mortar could have contributed to the collapse. Four material-dependent mechanisms are assessed—crushing, differential shortening between coursed masonry and monolithic shafts, time-dependent deformation under eccentric load, and the dependence of the buttressing on stone strength—using a traceable database that combines published tests on chalk representative of the Beauvais stone, data on air lime mortars, the documented geometry of the choir and field measurements of the masonry bond. Uncertainty is propagated through 10^5^ Monte Carlo simulations and expressed as central and characteristic safety factors and reliability indices. The central safety factor of the choir piers against crushing is 4.3 for the dry stone and 2.5 for the saturated stone, far below the margin implicit in earlier analyses. Coursed masonry shortens about 4 to 6 times more than a monolithic shaft in the long term, which, under strain compatibility, concentrates stresses of 2.5 to 5 times the mean value in the shafts. Creep-induced amplification is small in a laterally braced pier but becomes critical if lateral restraint is lost. The results indicate that the material did not cause the collapse through lack of strength but is compatible with a material contribution through its deformability and its sensitivity to water.

## 1. Introduction

On 29 November 1284, the high vaults of the choir of the Cathedral of Saint-Pierre in Beauvais partially collapsed, twelve years after their completion around 1272 [1]. The building, begun in 1225, reaches a maximum interior height of 48.50 m, the greatest ever achieved by a masonry vault in the Middle Ages. The subsequent reconstruction doubled the number of supports in the straight bays of the choir, which increased from three to six, and the building has kept that configuration ever since [1]. The episode is one of the most studied structural failures in historic architecture; yet, more than a century and a half after the first interpretations, there is still no consensus on its cause.

The proposed explanations can be grouped according to the role they assign to the material. Viollet-le-Duc attributed the collapse to a local detail: the failure of the monolithic colonnettes placed between the upper and lower flying buttresses, which carried an increasing load as the mortar of the adjacent pier dried and shrank [2,3]. Pihan and, later, Branner pointed to the excessive span of the bays and the slenderness of the supports [4,5]; Frankl pointed to the foundations [6]; Benouville defended the soundness of the original design and attributed the failure to faulty execution in the upper parts [7]. Heyman, applying the limit analysis he had himself formulated [8], concluded that the structure was globally stable, that the stresses in the masonry were very low compared with the strength of the stone, and that the failure must have been triggered by a local and somewhat trivial cause [3]. Wolfe and Mark, using a photoelastic model, identified a weakness at the base of the intermediate buttress uprights and pointed to wind as a relevant factor [9]. Murray, on the basis of an archaeological analysis of the fabric, located the origin of the collapse in the insufficient lateral buttressing of bay 7, where the outer buttress partly overhangs the outer aisle [1,10]. Taupin stressed the stabilising role of iron [11], and Como proposed a time-dependent buckling of the piers due to creep of the masonry [12].

This debate contains a methodological fracture that is rarely made explicit. The interpretations that give an active role to the material—those of Viollet-le-Duc, of Mainstone in his discussion of Heyman’s paper [3] and of Como [12]—attribute the damage to time-dependent processes: the shrinkage and slow hardening of lime mortar, and delayed deformation under eccentric loads. Mainstone also recalled that Goodyear had measured in 1903 a spread of almost one metre between the springings of the rebuilt vault [3], which shows that the geometry of the choir did not remain unchanged. Conversely, the tools with which these hypotheses were tested were, by construction, unable to represent such processes: Benouville’s graphic statics and Heyman’s limit analysis assume a fixed geometry and a rigid material of unlimited compressive strength [3,7,8], and Wolfe and Mark’s photoelastic model uses an elastic, linear and homogeneous material [9]. When Murray writes that Viollet-le-Duc’s hypothesis was “convincingly refuted” by Mark [1], he refers, strictly speaking, to a refutation obtained with a model that could not contain the proposed mechanism.

A second limitation is documentary. Heyman’s argument that the strength of the material is irrelevant relied on a comparison with generic values of stone strength [3], not on tests of the Beauvais stone. The later literature has not incorporated laboratory data on the fabric of the choir either. This gap also affects the author’s previous work on the building [13,14,15,16,17], which focused on its structural, historical and cultural interpretation and relied on dimensional and strength values taken from secondary sources.

The primary objective of this study is therefore to determine whether, and through which mechanisms, the properties of the stone and the lime mortar could have contributed to the 1284 collapse. To this end, four hypotheses are tested quantitatively against published data on the stone and the mortar:

**H1.**  *Crushing of the masonry.*

**H2.**  *Differential shortening between coursed masonry with lime joints and monolithic shafts laid against the bed (en délit).*

**H3.**  *Time-dependent deformation of the masonry under eccentric load.*

**H4.**  *The dependence of the bay 7 buttressing on the flexural strength of the stone.*

The novelty of the computational approach lies in three aspects. First, every input is linked to a documented source or declared as an explicit working assumption in a traceable database, so that the result of each hypothesis can be traced back to the data that control it. Second, instead of a single deterministic model of the whole structure, whose inputs would at present be largely conjectural, each mechanism is represented by a closed-form screening model and the uncertainty of all inputs is propagated by Monte Carlo simulation; the results are expressed as distributions, as central and characteristic safety factors and as reliability indices, which makes the strength of each conclusion explicit. Third, global and correlation-based sensitivity analyses identify which unmeasured properties govern the answer, turning the historical question into a testable research agenda for the material characterisation of the building. The analysis is extended to the cathedrals of Amiens and Reims: the former, built with a chalk of the same geological age [18], makes it possible to isolate the effect of geometry while keeping the material family constant; the latter, built with Lutetian limestone [19], provides a material contrast.

Beyond the historical question, the problem has practical relevance for the conservation of Gothic structures built with soft, porous stones and lime mortars. Assessment and strengthening decisions—repointing, moisture control, confinement of slender supports with inorganic-matrix composites such as fabric-reinforced cementitious matrix (FRCM) or textile-reinforced mortar (TRM) systems [20,21]—depend on the same properties examined here, namely the deformability of the joints, the time-dependent behaviour of the mortar and the sensitivity of the stone to water. The implications of the results for compatible intervention are discussed in Section 4.6.

## 2. Materials and Methods

### 2.1. The Choir of Beauvais: Geometry and Construction Sequence

The choir of Beauvais consists of three straight bays and a seven-sided hemicycle, surrounded by an ambulatory and seven radiating chapels [1]. The span of the central vessel, measured between pier axes, is 15.3 m [22]; Heyman adopted 15.0 m in his analysis [3]. The total length of the straight bays is 25.40 m [22], i.e., about 8.5 m per bay in the original configuration. The maximum interior height of the building, measured by the author with a laser distance metre in August 2024 at the centre of the cathedral, is 48.50 m, a value that coincides with that recorded by the French heritage inventory for the vault of the south transept arm [23]. For the vaults of the straight bays of the choir, Murray gives 46.75 m [1] (about 46.77 m according to the inventory [23]), and for the hemicycle vault about 46.3 m [22]. The models used in this study do not depend on the keystone height, but on the geometry of the supports and the loads they carry, so the difference between these values does not affect the results. The original main arcade piers have a cylindrical core 1.38–1.40 m in diameter, with two engaged columns on the longitudinal axis, and the main arcade wall is 1.34 m thick [1]. The main arcade reaches about 21 m, the same height as at Amiens, and the inner aisle vaults rise to about 21 m [1].

**Figure 1:**
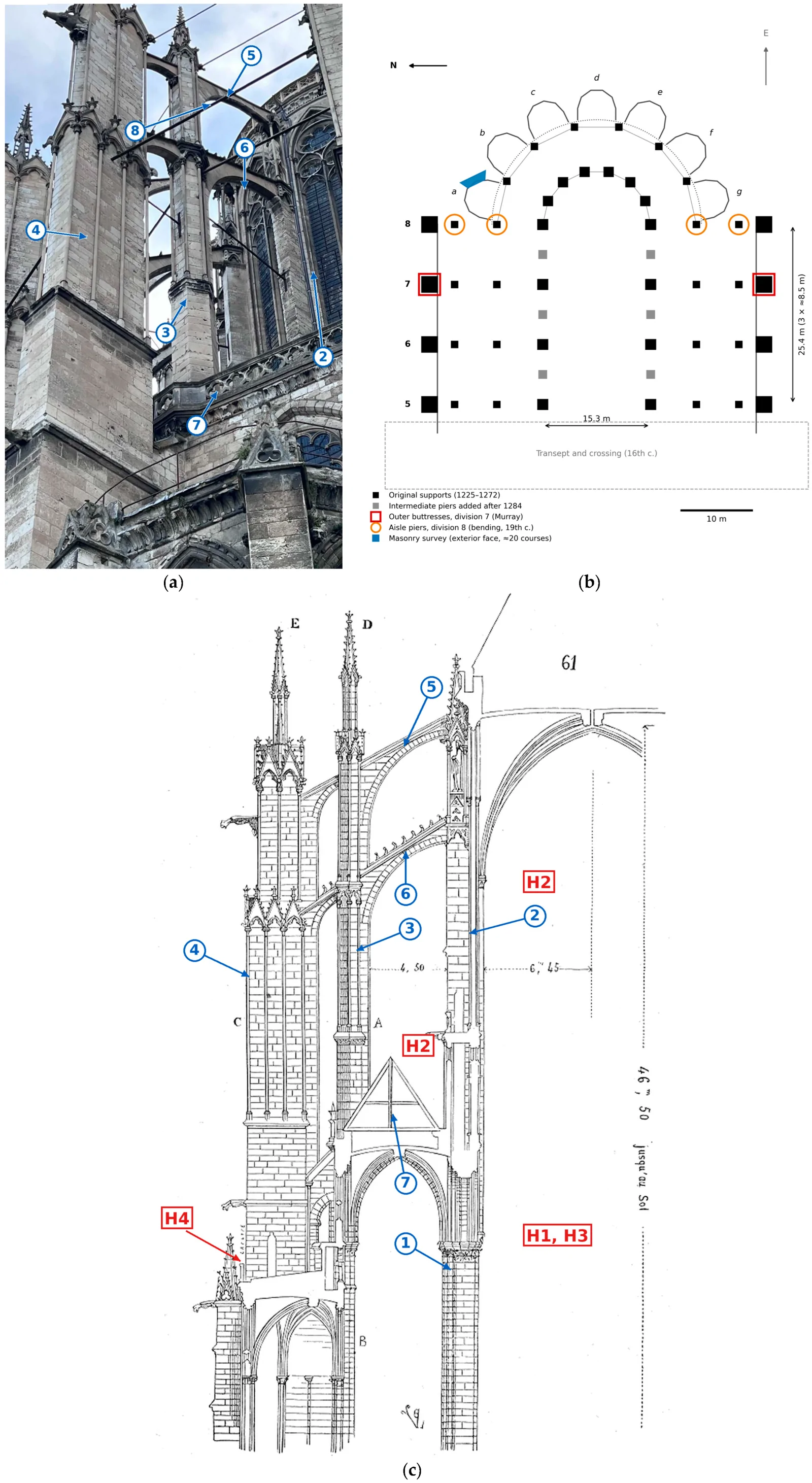
(**a**) Buttressing system of the hemicycle of Beauvais Cathedral, which survived the 1284 collapse (photograph by the author). (**b**) Schematic plan of the choir based on Murray [1,22], showing the original supports, the intermediate piers added after 1284, the outer buttresses of division 7, the aisle piers of division 8 and the location of the masonry survey (schematic, not a measured survey). (**c**) Transverse section of the choir by Viollet-le-Duc (Dictionnaire, vol. 1, “Arc-boutant”, Fig. 61; public domain [2]); letters A–E are Viollet-le-Duc’s own, and the dimensions in (c) keep his original notation with decimal commas. Numbers (**a**,**c**): 1, main pier of the choir; 2, clerestory wall and upper pier; 3, intermediate upright; 4, outer buttress upright; 5, upper flying buttress; 6, lower flying buttress; 7, ambulatory roof and terrace; 8, iron tie-rods. Red labels: Elements analysed in Hypotheses H1–H4. In (**b**), a–g denote the radiating chapels and 5–8 the divisions of the straight bays, after Murray [1].

Figure 1 presents the elements discussed in this study. Figure 1a shows the buttressing system of the hemicycle, which survived the 1284 collapse; Figure 1b locates the elements on a schematic plan; and Figure 1c reproduces the transverse section of the choir drawn by Viollet-le-Duc in the article “Arc-boutant” of his Dictionnaire [2], which shows the main pier, the clerestory wall and upper pier, the intermediate upright (Viollet-le-Duc’s pier A, set slightly out of line over the pier B that separates the two aisles), the outer buttress C, and the two levels of flying buttresses and the aisle roofs. The same numbers identify each element in the photographs and in the section, and the section indicates where each hypothesis acts. The drawing is Viollet-le-Duc’s interpretation, not a measured survey: it gives 46.50 m from the pavement to the vault, against the 46.75 m reported by Murray [1].

The chronology follows Murray [1]: works began in 1225, the high vaults of the choir were completed around 1272, the partial collapse occurred on 29 November 1284, and the reconstruction in the following decades added intermediate supports in the straight bays. The collapse of the crossing tower in 1573 lies outside the scope of this study.

The acting forces are taken from the classical analyses. According to the calculations of Benouville and Ungewitter reported by Heyman, the vault transmits to each half-bay a vertical load of about 36 t and a horizontal thrust of about 20 t, and wind on the roof adds about 16 t per bay [3]. Benouville’s graphic statics gives a vertical resultant of about 111 t at the level of the springing of the high vault (tas-de-charge), which includes the vault, the roof and the upper part of the support [3,7]. Benouville also reports a maximum stress of 13 kg/cm^2^ (≈1.27 MPa) in the masonry, assuming that the centring was removed after the mortar had set [3,7]; since the section to which this stress refers is not specified, it is used only as an order of magnitude and not to derive loads.

### 2.2. Materials

#### 2.2.1. Stone

**Figure 2:**
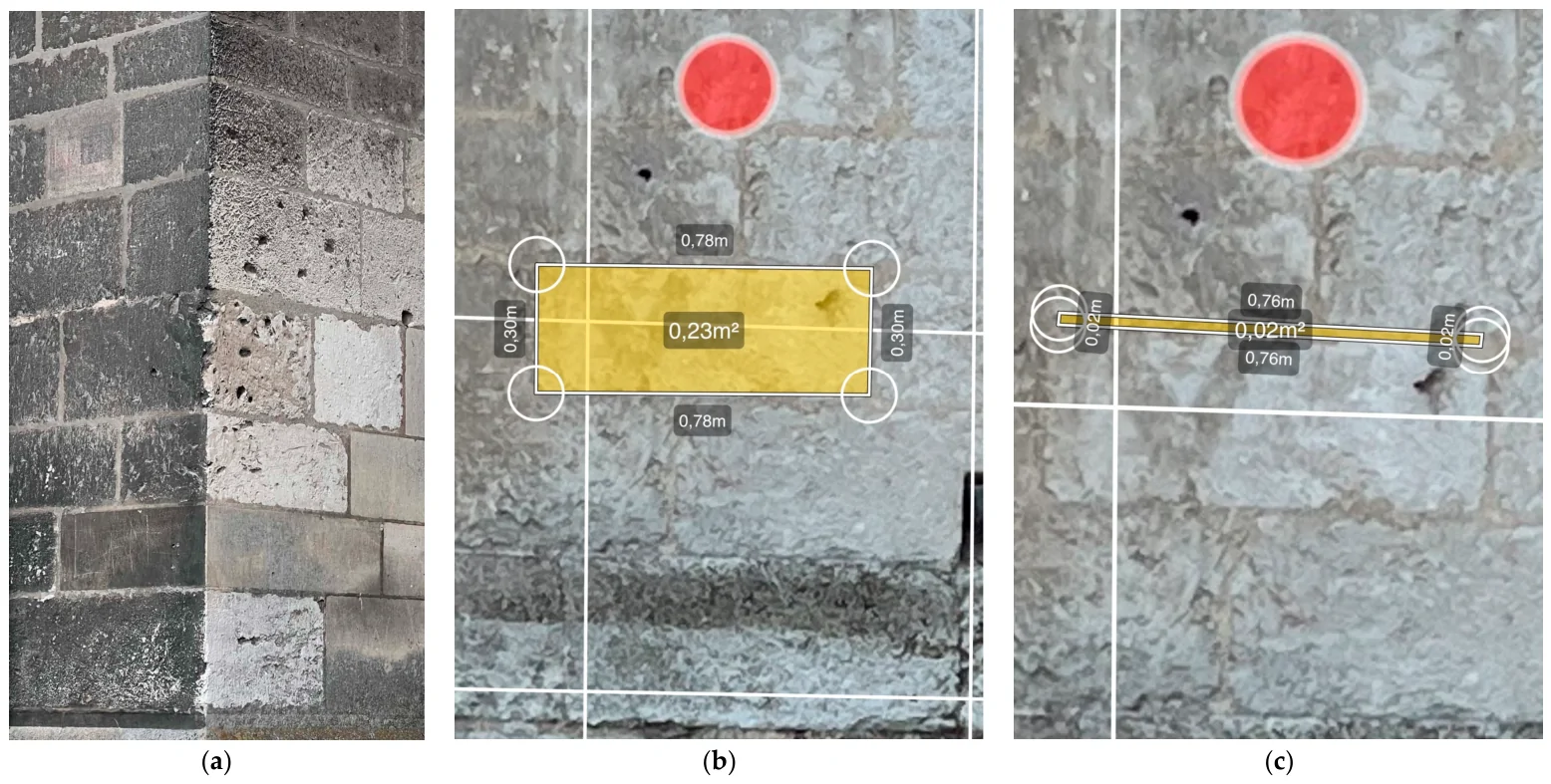

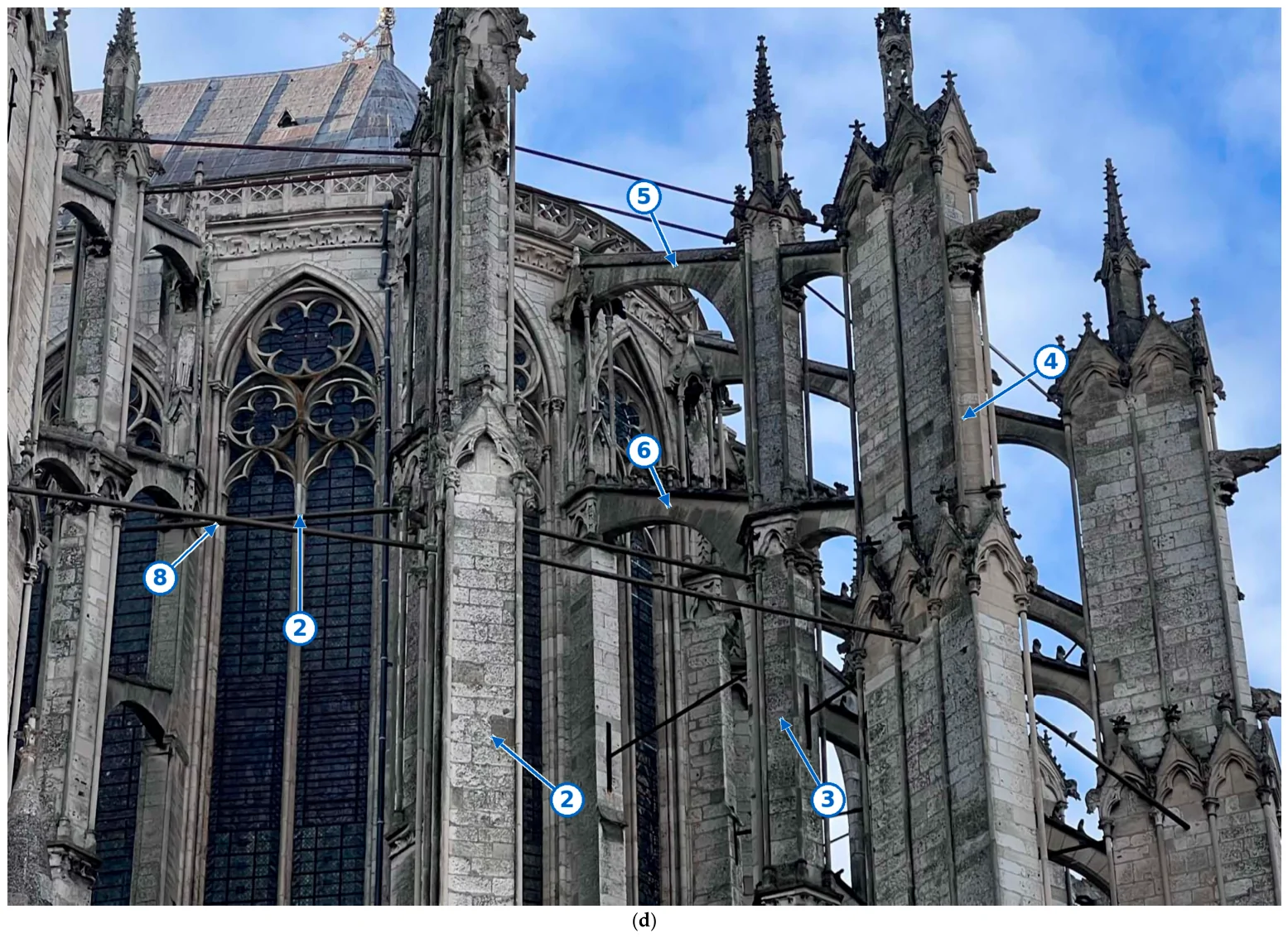
Masonry of the choir of Beauvais Cathedral. (**a**) Chalk ashlar of the lower zone, showing the variety of chalk qualities and the cavities left by detached flint nodules (photograph by the author). (**b**,**c**) Photogrammetric measurement on the exterior face of the north hemicycle chapel with a Stanley STHT1-77366 (Stanley Black & Decker, New Britain, CT, USA) device on an iPhone: (**b**) a 0.78 × 0.30 m block; (**c**) a 0.02 m joint along a 0.76 m bed (reading step 0.01 m). (**d**) Upper parts of the hemicycle, where the mechanisms of Hypotheses H2 and H4 act (photograph by the author). Numbers as in Figure 1: 2, clerestory window and upper pier with attached shafts en délit; 3, intermediate upright; 4, outer buttress upright; 5, upper flying buttress; 6, lower flying buttress; 8, iron tie-rod. The decimal commas in (**b**,**c**) are those displayed by the measuring application.

The stone of the choir is a Coniacian chalk. Murray describes it as a hard chalk with abundant flint nodules, used even in the slenderest exterior shafts of the upper choir, and distinguishes several qualities: a yellowish, harder chalk; a whiter, more weathered one; and a softer chalk reserved for the vault webs [1] (Figure 2a). According to the same author, the main medieval quarry was the so-called carrière Saint-Pierre, between Bongenoult and Frocourt, about 4 km south-east of the town [1].

The only published laboratory tests on a chalk of this type come from the study by BRGM and the Laboratoire de Recherche des Monuments Historiques on the selection of replacement stones [24]. The Craie de Picardie sample (reference M-CPI) is a block taken from the church of Saint-Étienne in Beauvais, from the Saint-Martin-le-Nœud quarries [24]. The tests followed the French standards then in force for limestones: total porosity and bulk and real density according to NF B 10-503 [25], water absorption coefficient according to NF B 10-504, capillary absorption according to NF B 10-502, and uniaxial compressive strength according to NF B 10-509 [26], on a first series of oven-dried specimens (*R_c_*) and a second series saturated by immersion at atmospheric pressure for 48 h (*R_c_*_48_) [24]. The report does not state the number, shape or size of the specimens, nor whether the dispersion that accompanies each value (±) is a standard deviation or a range; in this study it is interpreted as one standard deviation. The report does not give the bulk density of M-CPI either; from its total porosity of 34.9% and a calcite grain density of about 2.70 g/cm^3^, a dry bulk density of about 1.76 g/cm^3^ is estimated. The properties are summarised in Table 1. With a compressive strength of 7.7 MPa dry and 4.5 MPa after 48 h of immersion, it is the second weakest of the eighteen stones in the study in the wet state, and its strength loss on saturation (42%) exceeds the average of the set (30%) [24].

The M-CPI sample is a geological proxy, not a sample of the cathedral fabric: it comes from another quarry of the same formation, not from the main quarry identified by Murray, and Murray’s description of the choir stone as a “hard chalk” suggests that the best beds used in the supports may have been stronger than the proxy. This uncertainty is incorporated explicitly through two stone scenarios (Section 2.6). Scenario A uses the proxy as tested, *R_c_*~*N*(7.7, 0.8) MPa. Scenario B is a proxy envelope in which *R_c_* is log-uniform between 6.2 MPa (the M-CPI mean minus two standard deviations) and 20 MPa, the lower half of the range of 15–40 MPa reported for selected medieval building chalkstones of Normandy [27]; in scenario B the saturated strength is obtained by applying to each sampled value the M-CPI saturation ratio, *R_c_*_48_/*R_c_*~*N*(0.584, 0.05). The wider ranges reported for chalks of the Anglo-Paris Basin—dry compressive strengths as low as 3.5–4 MPa in coastal chalk and losses of 40–50% in strength and modulus on wetting [28]—indicate the variability of the lithological family.

The cathedral of Amiens was built in its first phase (1220–1234) with a Lower Coniacian chalk from the Croissy-sur-Celle area [18], i.e., with a material of the same family as that of Beauvais. The cathedral of Reims was built with Lutetian limestone, whose reference quarry is Courville; the porosity of its beds ranges from 13% to over 50% depending on the level [19]. Neither source reports mechanical tests, so the comparison with Amiens and Reims is lithological and geometrical rather than quantitative in terms of strength.

#### 2.2.2. Mortar

No published characterisation of the original mortar of the choir has been found. Reference values for air lime mortars are therefore adopted, with the caveat that they come from modern specimens. Drougkas, Roca and Molins tested masonry with an air lime mortar CL90 (1:3, without cement) and obtained, for the mortar, a compressive strength of 1.23 MPa at 49 days and an elastic modulus of about 125 MPa, derived through a series homogenisation model [29]. The RILEM TC 277-LHS report places the 28-day compressive strength of air lime mortars between 0.5 and 2.0 MPa, with open porosities of 23–40% [30].

The value of 125 MPa corresponds to a young mortar and is adopted as the lower bound of stiffness. The hardening of air lime is controlled by the diffusion of CO_2_ from the exposed faces: carbonation fronts advance quickly near the joint surface but slowly in depth, and strength and stiffness continue to increase for months or years after laying [31,32]. Drougkas et al. observed that the centre of the joints of their specimens had not fully carbonated after 49 days [29], and carbonates formed long after construction have been documented in historic mortars [33]. In the outer few centimetres of the joints, carbonation would have been essentially complete within the twelve years between the completion of the vaults and the collapse; in the inner mortar of solid piers 1.4 m in diameter and of thick walls, it may have remained partial. This is a plausibility argument, not a measured fact, and the range of the modulus is set so that both states are covered.

For carbonated mortar, an upper bound of 500 MPa is adopted, justified on three grounds. First, it is four times the modulus of young air lime and twice the value obtained for a natural hydraulic lime mortar NHL 3.5 in the same test (250 MPa) [29], so it allows for considerable hardening without attributing to the medieval mortar a stiffness typical of a hydraulic binder. Second, air lime mortars with ceramic additions, which have a pozzolanic character, reach moduli of about 660 MPa, values regarded in the literature as higher than those of typical medieval air lime mortars [34]; the adopted bound lies below that figure. Third, the gain in stiffness with carbonation is progressive and the bulk of the joint volume in the piers is far from the exposed surface [31,32]. The interval from 125 to 500 MPa thus spans from freshly hardened to largely carbonated mortar, and within it the modulus is treated as a random variable. Because the upper bound is a working assumption, the analysis is repeated with upper bounds of 1000 and 2000 MPa, and the dependence of the results on a fixed modulus between 125 and 3000 MPa is reported (Section 3.3).

The creep coefficient of the mortar, φ, is not known for lime mortars of this age. A uniform range of 0.5–3 is adopted as a working assumption: its lower part corresponds to the final creep coefficients given by EN 1996-1-1 for masonry (0.5–2.0, depending on the unit type) [35], and its upper part allows for the higher creep of young, partially carbonated lime mortar. Because stiffer, more carbonated mortars are also expected to creep less, a negative correlation between Em and φ is examined in the sensitivity analysis.

#### 2.2.3. Joints and Vertical Gradient of the Masonry

Two independent observers, a century apart, describe a variation in the quality of the masonry with height. Benouville notes that the bond is very careful up to the triforium and much less so above, where he suspects the use of leftover pieces [7]. Murray observes that the ashlar of the lower parts is yellowish with narrow joints, whereas above the ambulatory roof the stone is whiter and the joints wider and more irregular [1]. Murray also reports a reduction in course height in the upper part of the walls [1]. Neither author provides measurements.

For the lower zone, original measurements by the author are available for about twenty courses of the exterior face of the chapel wall on the north side of the hemicycle, near the first window (Figure 1b). They were made by photogrammetric rectification using a Stanley STHT1-77366 digital measuring device on an iPhone (Apple Inc., Cupertino, CA, USA) with the Stanley Smart Measure Pro application (Figure 2b,c). The application reports lengths in steps of 0.01 m; course heights and block lengths, of 0.30 m and 0.76–1.19 m, are therefore known to within about ±3% and ±1%, respectively, whereas the joint thickness, which is of the same order as the reading step, is strongly quantised: a reading of 0.02 m, the maximum recorded, corresponds to a true thickness between 15 and 25 mm, and a reading of 0.01 m to 5–15 mm. The measurement is therefore used only to bound the joint thickness, not to estimate its distribution. Because the face is exterior and weathered, and the erosion of the chalk arrises widens the mouth of the joints, the readings are also interpreted as an upper bound of the true bed thickness. A range of 5–20 mm for the joint thickness and 280–320 mm for the course height is therefore adopted for the lower zone, and the effect of the quantisation is tested by extending the range to 5–25 mm.

No measurements are available for the upper zone (Figure 2d). Following the descriptions of Benouville and Murray, a working assumption of 15–30 mm for the joint thickness and 250–400 mm for the course height is adopted. The influence of these ranges on the results is assessed explicitly in the sensitivity analysis. It should also be noted that the hemicycle chapels belong to the first building campaign, begun in 1225 [1], whereas the piers of the straight bays and the upper choir were built in later campaigns. The measurements are therefore representative of the fabric of the building, but not directly of the supports that failed in 1284.

#### 2.2.4. Iron

Iron elements in the fabric of Beauvais are documented by archaeometric analyses and radiocarbon dating, which make it possible to distinguish between reinforcements planned in the initial building phases and later consolidations [36,37]. Their role is considered in the Discussion but is not modelled quantitatively.

### 2.3. Database and Source Selection Criteria

All parameters have been gathered in a database (Supplementary Material, Table S1) in which each value is recorded with its source, unit and, where possible, range or standard deviation. Three criteria were applied. First, primary sources prevail: geometric values are taken from direct studies of the building [1,22] or from the author’s own measurements, not from later syntheses; when two primary sources disagree, both are recorded and a range is adopted. Second, tests prevail over citations: material properties come from documented laboratory tests [24,29,30]; values without an identifiable test are excluded. Third, assumptions are stated explicitly: parameters without direct experimental support—upper-zone joint geometry, creep coefficient, upper bound of the mortar modulus and the load components estimated from volumes—are identified as working assumptions and treated as random variables in the uncertainty analysis.

### 2.4. Axial Load on the Choir Pier

The axial load N at the base of a choir pier is built up from six components (Table 2). The upper part is anchored on the only documented resultant, Benouville’s 111 t at the level of the tas-de-charge, at 40.25 m (46.75 m minus the 6.5 m rise of the vault [1,3]); a range of 105–111 t is adopted to allow for the reading uncertainty of the graphical construction. Below that level the components are computed from volumes taken from Murray’s dimensions [1] and a masonry density ρ between 1.76 t/m^3^ (the dry chalk, Section 2.2.1) and 2.00 t/m^3^ (allowing for mortar, flint and denser beds): the upper pier with its engaged shafts and the adjoining wall strips between the tas-de-charge and the triforium sill at 21 m; the tracery, glazing and triforium passage; the main arcade spandrels, computed for a wall 1.34 m thick and a bay 8.5 m long between the capital and 21 m, minus a pointed-arch opening; the share of the inner aisle vaults and roof; and the self-weight of the pier up to the capitals. Each component is sampled independently in every simulation from the distribution in Table 2, and N is their sum. The resisting area A at the base is that of the cylindrical core plus the two engaged columns, 1.60–1.80 m^2^.

### 2.5. Testing Models

**Figure 3:**
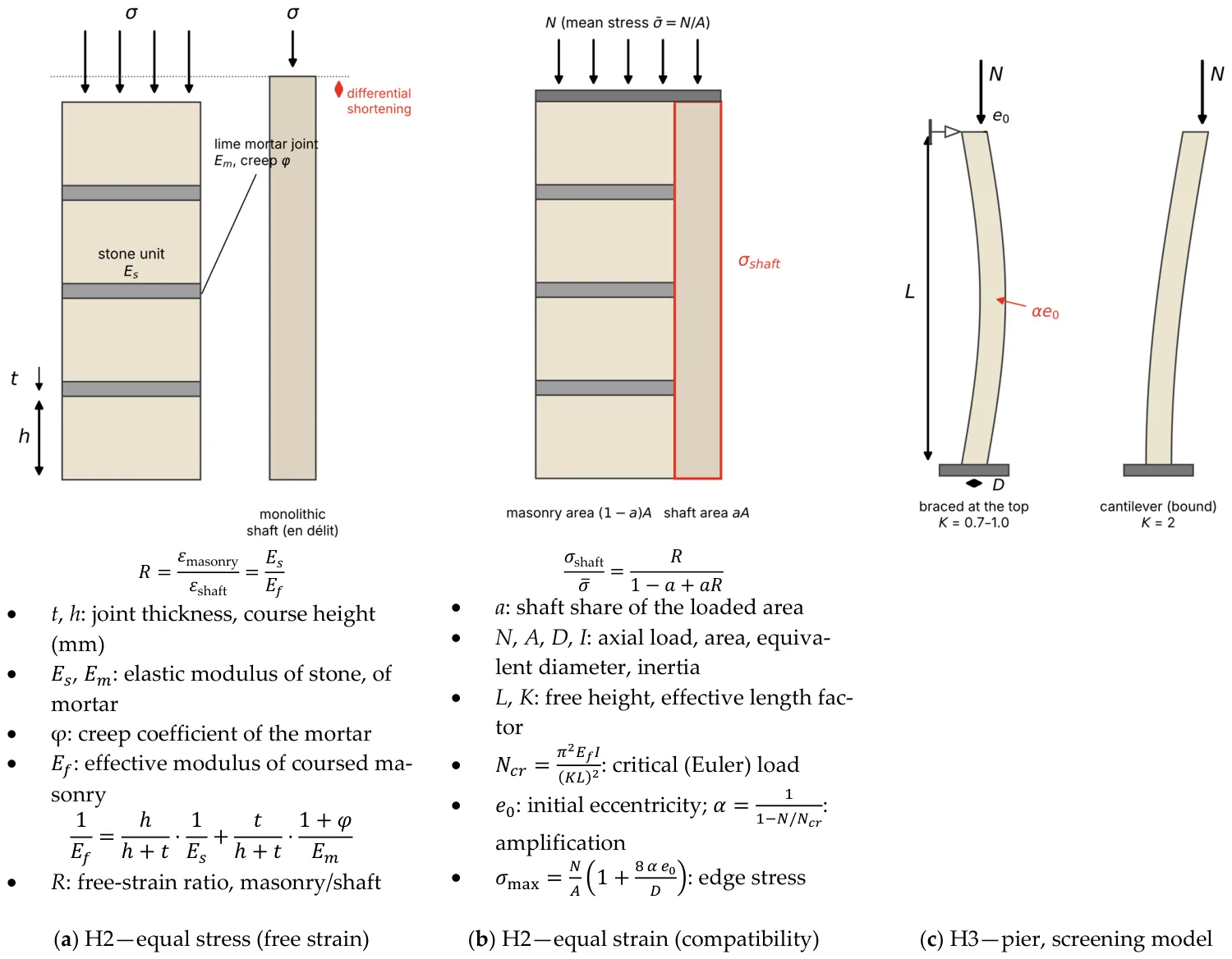
Schematic of the models and nomenclature. (**a**) H2, equal-stress limit: coursed masonry with units of height h and lime mortar joints of thickness t, and a monolithic shaft laid en délit, under the same stress σ; the masonry shortens more than the shaft (R=EsEf>1). (**b**) H2, equal-strain limit: when both elements share a common bearing, compatibility forces the same shortening and the shaft, with area fraction a, carries a higher stress σ_shaft_. (**c**) H3: pier of free height *L* and equivalent diameter *D* under an axial load *N* with initial eccentricity e0, amplified to α·*e*_0_ by second-order effects, for a pier braced at the top (*K* = 0.7–1.0) and for the cantilever bound (*K* = 2). Joint thickness is exaggerated for clarity.

Each hypothesis is tested with a deliberately simple closed-form model with traceable parameters. This choice does not reflect a lack of means but the nature of the question: the aim is to establish whether the orders of magnitude imposed by the materials are compatible with each mechanism, not to reproduce the collapse with a numerical model whose inputs would, in the present state of knowledge, be mostly assumptions. The models are therefore screening tools: they do not represent cracking, contact or the three-dimensional interaction of the structure, and their results indicate whether a mechanism is plausible with the documented materials, not whether it occurred. The models for H2 and H3 and all the variables they use are shown in Figure 3.

#### 2.5.1. H1: Crushing Criterion

The reference stress in the masonry is compared with its strength. The reference stress is the mean stress at the base of the choir piers, computed as described in Section 2.4. Strength is evaluated at three levels: that of the dry stone (*R_c_*) and the saturated stone (*R_c_*_48_) according to BRGM [24], and that of the masonry, estimated with the expression of EN 1996-1-1 [35]:

(1) $$f_{k}=K\;f_{b}^{0.7}\;f_{m}^{0.3},\;\;K=0.45$$

EN 1996-1-1 is used because it is the only codified, widely validated relationship between unit strength, mortar strength and the strength of unreinforced masonry, and it explicitly covers dimensioned natural stone units (Group 1), for which, with general purpose mortar, *K* = 0.45 [35]. The stone strength is converted into the normalised mean compressive strength fb required by the code following EN 772-1 [38]: *f_b_* = 0.8·δ·Rc, where 0.8 is the conditioning factor that converts the strength of oven-dried specimens to the air-dry reference state, and δ is the shape factor that converts the strength of the test specimen to that of a 100 mm cube; since the specimen size is not reported, δ is treated as uniform between 0.80 and 0.95, the values of EN 772-1 for small specimens. The mortar strength *f_m_* is taken as 1.23 MPa [29]. As *f_k_* is a characteristic (5%) value, the corresponding mean strength is estimated as *f_mean_* = 1.2 *f_k_*, in accordance with EN 1052-1 [39], and this mean value is used in the central safety factor.

The code expression is used as a lower bound, for three reasons. It was calibrated mainly on manufactured units with general purpose mortar joints of 6–15 mm [35], whereas the Beauvais masonry consists of large ashlar blocks, 0.30 m high and up to 1.2 m long, in which the joint occupies only 2–6% of the height; for such masonry the strength approaches that of the units. Drougkas et al. showed that it underestimates the strength of masonry with air lime mortar [29]. And the collapse of 1284 did not begin with the crushing of the choir piers, which is itself evidence that the actual strength exceeded the code value (Section 3.2). The ratio between strength and stress is compared with the margin implicit in Heyman’s reasoning [3], which follows from comparing Benouville’s stress with a stone strength of at least 150 kg/cm^2^ (≥11.5).

#### 2.5.2. H2: Differential Shortening Between Coursed Masonry and Monolithic Shaft

A portion of a support is considered in which coursed masonry, made of units of height h and mortar joints of thickness t, works alongside a monolithic shaft laid en délit, without intermediate joints. Treating the masonry as two springs in series—the same homogenisation scheme used by Drougkas et al. to derive the mortar modulus [29]—the effective modulus of the coursed masonry, *E_f_*, is given by

(2) $$\frac{1}{E_{f}}=\frac{h}{h+t}\cdot \frac{1}{E_{s}}+\frac{t}{h+t}\cdot \frac{1+\varphi }{E_{m}}$$

where *E_s_* and *E_m_* are the elastic moduli of stone and mortar and *φ* is the creep coefficient of the mortar; with *φ* = 0 the instantaneous value is obtained, and with *φ* > 0 the long-term value. Two limiting states bracket the real behaviour (Figure 3a,b). In the equal-stress limit, both elements carry the same stress and shorten independently; the ratio between the strain of the masonry and that of the shaft is

(3) $$R=\frac{h}{h+t}+\frac{t}{h+t}\cdot \frac{E_{s}}{E_{m}}\;\left(1+\varphi \right)$$

*R* = 1 indicates compatible strains; *R* > 1 indicates that the masonry tends to shorten more than the shaft. This limit measures the incompatibility, but it is not a stress state that can persist where the two elements share a bearing: as soon as differential shortening develops, the load is redistributed towards the stiffer element. In the equal-strain limit, full compatibility is enforced; if the shaft occupies a fraction a of the loaded area, equilibrium and compatibility give the stress in the shaft relative to the mean stress $\bar{\sigma }$ = *N*/*A*:

(4) $$\frac{\sigma_{\mathrm{shaft}}}{\bar{\sigma }}=\frac{R}{1-a+\mathrm{aR}}$$

The real behaviour lies between the two limits and depends on the stiffness of the bearings, the sequence of loading and the creep history, which are not known; the equal-strain limit is conservative for the shaft. It is evaluated for *a* = 0.05, 0.10 and 0.20, a range that covers slender attached shafts and the monolithic colonnettes of the upper supports described by Viollet-le-Duc. Using the long-term Ef in this expression assumes that the redistribution caused by creep has fully developed, which is again an upper bound. This is the mechanism described by Viollet-le-Duc [2,3]. The model is evaluated separately for the lower and upper zones defined in Section 2.2.3.

The modulus of the stone has not been measured on the Beauvais chalk. It is estimated as *E_s_* = MR·*Rc*, where MR is the modulus ratio, for which a range of 500–1500 is adopted. Hoek and Diederichs place chalks at the high end of this ratio [40], and values above 1000 have been reported for high-porosity chalks owing to their low strength [41]. With the mean strength of the M-CPI chalk, the resulting modulus ranges from about 4 to 12 GPa.

#### 2.5.3. H3: Second-Order Model of the Pier

The choir pier is analysed as a slender support under an axial load *N* with initial eccentricity *e*_0_ (Figure 3c). As a screening model, the compound section (core plus engaged columns) is replaced by an equivalent homogeneous circular column of the same area *A*, with diameter D=4Aπ=1.43−1.51 m and moment of inertia I=πD464. The elastic modulus of this column is the effective modulus of the coursed masonry, Ef, defined in Section 2.5.2: it represents the stone and the joints as a homogenised material whose stiffness decreases with time as the mortar creeps. The Euler critical load is Ncr=π2EfIKL2, where *L* is the free height and *K* the effective length factor, and the eccentricity amplification factor is α=11−NNcr. The maximum edge stress is

(5) $$\sigma_{\max}=\frac{N}{A}\left(1+\frac{8\;\alpha \;e_{0}}{D}\right)$$

valid while the resultant remains within the core of the section (α e0≤D8). Beyond it, the section cracks partially, since the masonry has no tensile strength, and a damage state is considered to be reached. The model expresses in elementary form Como’s conjecture [12] and Mainstone’s objection [3]: the pier does not fail because of the mean strength of the material, but because its stiffness decreases with time while the eccentricity grows.

Two boundary conditions are considered. In the reference case, the pier is laterally braced at the level of the capitals by the main arcades, the aisle vaults and the transverse arches, and *K* is taken between 0.7 (partial rotational restraint) and 1.0 (pinned ends). The cantilever case, *K* = 2, is not a description of the original structure but a bound: it represents a pier whose top is free to sway, as would happen if the lateral restraint provided by the superstructure and the buttressing were lost or became ineffective. The cantilever bound is included because the effectiveness of that restraint is precisely what Murray’s explanation of the collapse calls into question [1,10]. The free height up to the arcade capitals is estimated at 14–16 m, below the 21 m of the arcade and inner aisle vaults reported by Murray [1], and the initial eccentricity at 20–100 mm. The approach is a screening model: an equivalent homogeneous column with Euler amplification does not represent cracking, the non-linear stress–strain behaviour of the masonry or the interaction with the rest of the structure, and its results indicate the sensitivity of the pier to lateral restraint rather than its actual capacity.

#### 2.5.4. H4: Dependence of the Buttressing on the Flexural Strength of the Stone

Murray’s explanation places the origin of the collapse in bay 7, where the outer buttress partly overhangs the outer aisle [1,10] (Figure 1b,c). Murray himself observes that this arrangement only works if the units at its base are wide enough to act as corbels and that, elsewhere in the building, smaller units gave way and loaded the arch below [10]. This hypothesis is assessed qualitatively, since the flexural and tensile strengths of the Beauvais chalk have not been measured [24].

### 2.6. Uncertainty, Reliability and Sensitivity Analysis

The complete set of input distributions is given in Table 3. Stone strength is modelled with a normal distribution with the mean and standard deviation of the BRGM test [24] in scenario A and with the log-uniform envelope described in Section 2.2.1 in scenario B. Parameters bounded by an interval without further information—joint thickness, course height, MR, creep coefficient, load components, free height, *K* and *e_0_*—are modelled with uniform distributions, which assign equal weight to the whole interval and are therefore conservative with respect to the tails. The mortar modulus, which varies over almost an order of magnitude between the young and the carbonated state, is modelled with a log-uniform distribution between 125 and 500 MPa. For each hypothesis and zone, 10^5^ Monte Carlo simulations with a fixed seed (1284) are run, and the 5th, 50th and 95th percentiles of each result are reported. In each simulation all inputs, including every load component and every strength, are sampled once; the load effect and the resistance are then computed for that same sample.

The inputs are treated as statistically independent, because no data exist to quantify their dependence. Two physically motivated correlations are nevertheless plausible and are tested explicitly with a Gaussian copula: a negative correlation between the mortar modulus and its creep coefficient (more carbonated mortars are stiffer and creep less), with ρ(*E_m_*, φ) = −0.5 and −0.8; and a negative correlation between stone strength and modulus ratio, ρ(*R_c_*, *MR*) = −0.5, reflecting the tendency of weaker, more porous chalks to have higher modulus ratios [41].

The safety against crushing is expressed by four complementary measures, computed from the simulated resistance *R* (*R_c_*, *R_c48_* or fmean) and load effect S (the mean stress σ, or σ_max_ in H3). The central safety factor is the ratio of the means γc=μRμS. The characteristic safety factor is γk=R5%S95%, the ratio between the 5th percentile of the resistance and the 95th percentile of the load effect, which is closer to the format of design codes. The reliability index is computed, following EN 1990 [42], for normally distributed variables, βN=μR−μSσR2+σS2, and, as a check, for log-normal variables, βLN=lnμRμSVR2+VS2, where *V* is the coefficient of variation. Finally, the probability *P_f_* = *P*(*R* ≤ *S*) is estimated directly from the simulations, with β*_MC_* = −Φ^−1^(*P_f_*); with 10^5^ simulations, *P_f_* can be resolved down to about 10^−5^ (β*_MC_* ≈ 4.3). The target value β = 3.8 of EN 1990 for a 50-year reference period [42] is quoted only as a familiar reference: it is calibrated for the design of new structures and not for the assessment of a medieval building.

The output distributions differ in shape for a structural reason. The safety factors of H1 are ratios between nearly normal resistances and a load that is the sum of several independent components, and they are therefore nearly symmetric. The stability and deformation results—*R* in H2 and α in H3—contain the mortar modulus, which is log-uniform, in the denominator, and α also contains the non-linear term 11−NNcr, which grows without bound as *N* approaches *N_cr_*; these outputs are therefore strongly right-skewed and resemble log-normal distributions.

The influence of each parameter is measured with Spearman’s rank correlation coefficient between input and output. The Python 3.13 code (NumPy 2.5, SciPy 1.18, Matplotlib 3.11) is provided as Supplementary Material (Code S1) and reproduces every number reported in Section 3.

### 2.7. Use of Generative Artificial Intelligence

During the preparation of this work, the author used Claude (Claude Opus 5.5; Anthropic, San Francisco, CA, USA) to locate bibliographic sources, extract data from tables in scanned documents, write the calculation scripts and assist with the drafting and translation of the text. The author has reviewed and verified all data, code and text and takes full responsibility for the content of this publication.

## 3. Results

### 3.1. Loads and Stresses at the Base of the Choir Piers

The estimated axial load at the base of the choir piers is 309 t (5th–95th percentile: 288–331 t), which produces a mean stress of 1.78 MPa (1.64–1.94 MPa) on the core and engaged columns (Table 2). The largest contributions are the resultant at the tas-de-charge (35%), the arcade spandrels (22%) and the upper pier between 21 m and 40.25 m (16%). The stress is higher than Benouville’s 1.27 MPa, as expected for the base of the support.

### 3.2. H1: Crushing

**Figure 4:**
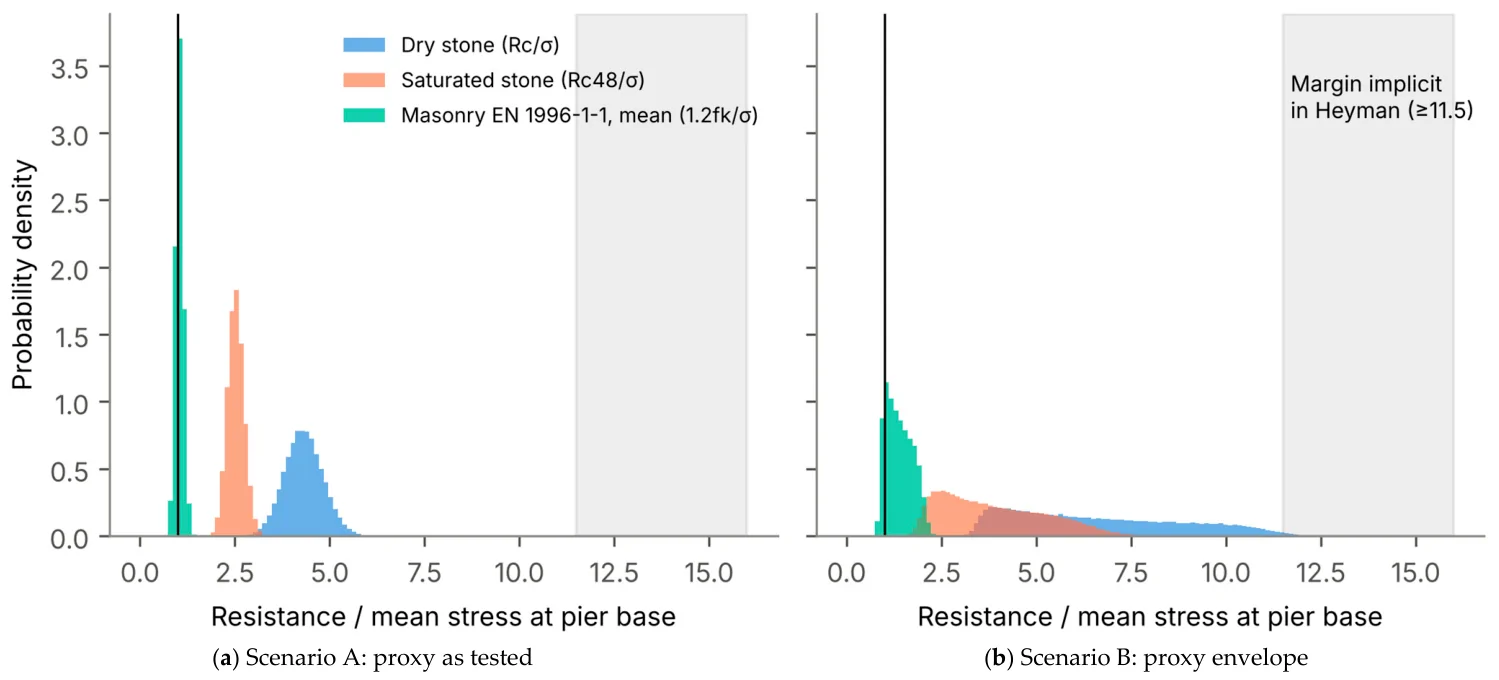
Ratio between resistance and mean stress at the base of the choir piers (10^5^ simulations): (**a**) scenario A, stone proxy as tested; (**b**) scenario B, proxy envelope. Dry stone, saturated stone and mean masonry strength according to EN 1996-1-1. The vertical line marks a ratio of 1; the grey band indicates the margin implicit in Heyman’s reasoning [3].

The safety factors against crushing are summarised in Figure 4 and Table 4, and the reliability measures in Table 5. In scenario A, referred to the dry stone, the ratio between strength and mean stress is 4.31 (3.51–5.16); referred to the saturated stone, 2.52 (2.19–2.88). In scenario B, which allows the cathedral stone to be stronger than the proxy, the medians rise to 6.25 and 3.64, with a much wider spread. In all cases they are far below the margin of at least 11.5 implicit in Heyman’s reasoning [3].

Referred to the mean strength of the masonry estimated with EN 1996-1-1, the ratio falls to 1.04 (0.89–1.22) in scenario A and 1.36 (0.92–1.98) in scenario B, and the probability that the code strength is exceeded is 0.33 and 0.13, respectively. These values must be read as a demonstrably conservative lower bound rather than as a prediction: the choir piers did not crush in 1284, and the piers of the hemicycle, unaffected by the reconstruction, have carried stresses of a similar order for more than seven centuries, which is incompatible with a probability of failure of one third. The result confirms that the code expression, calibrated for units and joint proportions unlike those of ashlar masonry, underestimates the strength of this masonry, as Drougkas et al. already noted for lime masonry [29]. The actual margin of the interior piers, for which the dry state is the relevant one, therefore lies between the code bound and that of the stone.

In terms of reliability, the dry and saturated stone give central safety factors of 4.3 and 2.5 (scenario A), no failure in 10^5^ simulations (β*_MC_* > 4.3) and normal reliability indices β*_N_* of 7.3 and 8.6, well above the reference value of 3.8. In scenario B the central factors are higher (6.6 and 3.9), but β*_N_* falls to 2.5 and 2.1 because the wide log-uniform envelope inflates the standard deviation of the resistance; the log-normal index β*_LN_* (5.6 and 3.9) and the direct simulation (no failures) show that this is an artefact of the normal assumption applied to a skewed distribution, not a lack of safety.

### 3.3. H2: Differential Shortening

**Figure 5:**
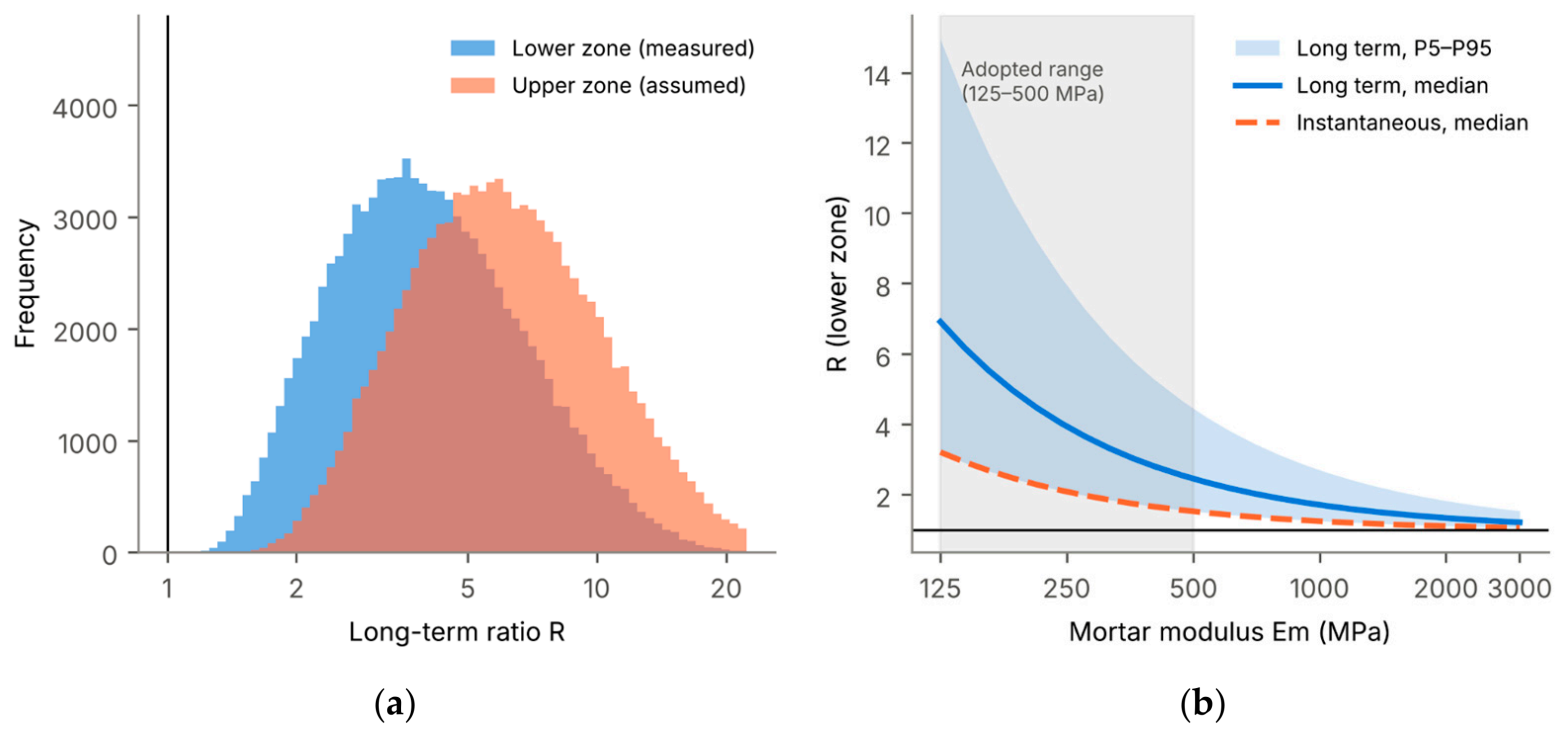
(**a**) Ratio *R* between the long-term shortening of coursed masonry and that of a monolithic shaft, in the lower (measured) and upper (assumed) zones. (**b**) Dependence of *R* (lower zone) on a fixed mortar modulus: median and 5th–95th percentile band in the long term and median of the instantaneous value; the grey band marks the adopted range of 125–500 MPa.

In the lower zone, with joints of 5–20 mm and courses of 280–320 mm, coursed masonry shortens instantaneously 2.07 times more than a monolithic shaft (1.35–4.00) and, in the long term, 3.91 times more (1.88–9.78). In 93% of the simulations the long-term ratio exceeds 2. In the upper zone, with the assumed joints of 15–30 mm, the values rise to 2.81 (1.67–5.60) instantaneously and 5.94 (2.69–14.69) in the long term, and the ratio exceeds 2 in 99% of cases (Figure 5a, Table 6).

Under the equal-strain limit, this incompatibility becomes a stress concentration in the shaft. For a slender shaft occupying 5% of the loaded area, the long-term stress in the shaft is 3.4 (1.8–6.8) times the mean stress in the lower zone and 4.8 (2.5–8.7) times in the upper zone; for a = 0.20 the concentration falls to 2.5 (1.6–3.6) and 3.0 (2.0–3.9), respectively (Table 6). These factors are of the same order as the safety factors of the stone obtained in H1 (2.5–4.3 in scenario A): where a shaft works in compatibility with coursed masonry, the redistribution caused by the mortar alone can consume most or all of the margin of the shaft.

In both zones the most influential parameter is the mortar modulus (Spearman coefficient −0.58 in the lower zone and −0.64 in the upper zone), followed by the joint thickness (+0.51 in the lower zone, where it is a measured value with a relatively wide range), the stone modulus (+0.46 and +0.50) and the creep coefficient (+0.38 and +0.42). Course height has a minor influence.

The sensitivity analysis (Table 7, Figure 5b) shows that the qualitative result is robust but its magnitude depends strongly on the mortar modulus. Extending the lower-zone joints to 25 mm to allow for the quantisation of the readings raises the median from 3.91 to 4.41. A stronger and stiffer stone (scenario B) increases *R*, so the proxy is conservative for H2. The correlations between Em and φ and between *Rc* and MR leave the medians practically unchanged and only widen the upper tail. Raising the upper bound of the mortar modulus to 1000 or 2000 MPa reduces the median in the lower zone to 3.03 and 2.41. For a fixed modulus (Figure 5b), the long-term median falls from 6.9 at 125 MPa to 3.0 at about 360 MPa, 1.7 at about 1 GPa and 1.2 at 3 GPa: the mechanism is significant for a young or partially carbonated air lime mortar and fades for a stiff, fully carbonated or hydraulic one.

### 3.4. H3: Time-Dependent Deformation with Eccentricity

**Figure 6:**
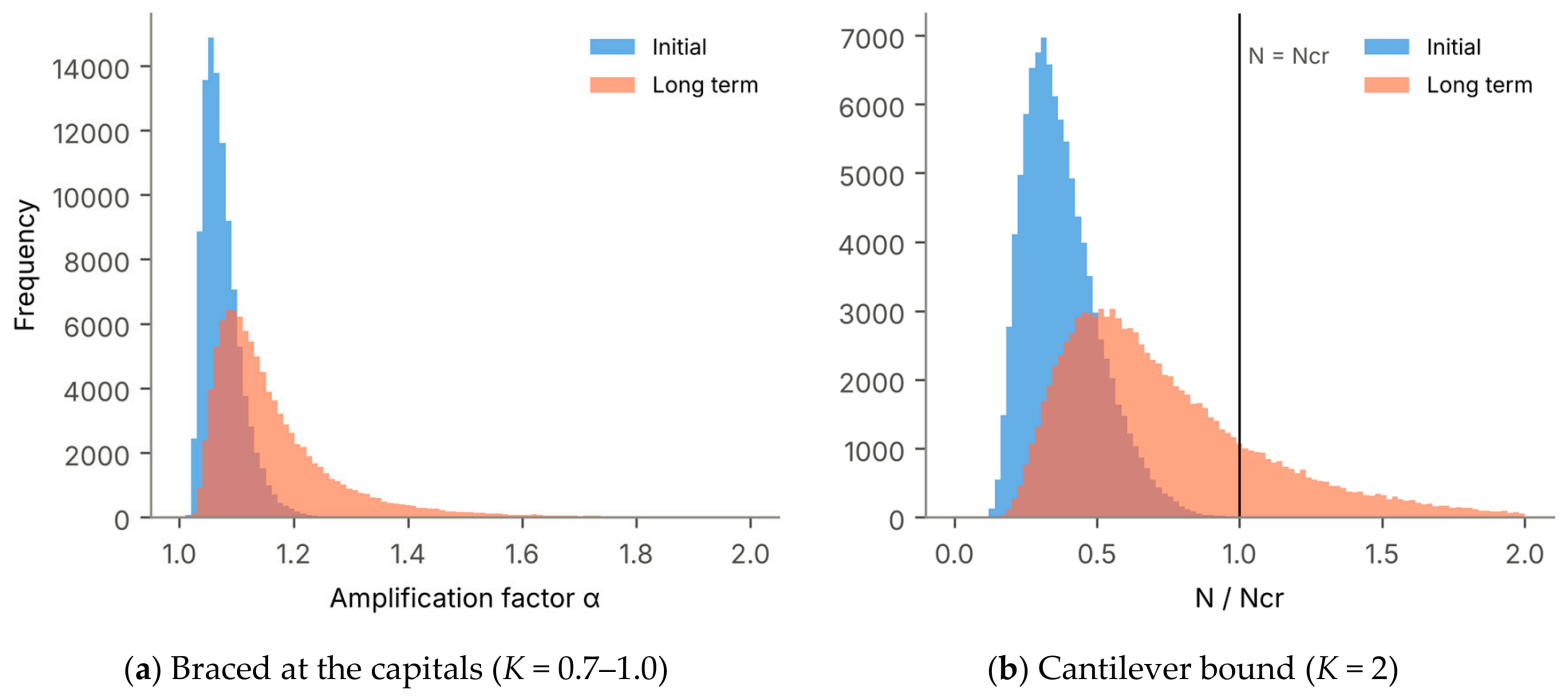
Second-order effects in the choir pier, initially and in the long term (scenario A): (**a**) eccentricity amplification factor α for a pier braced at the capitals (*K* = 0.7–1.0); (**b**) ratio NNcr for the cantilever bound (*K* = 2); the vertical line marks the critical load.

For the braced pier, the effective modulus of the masonry is 3.4 GPa (2.0–5.8 GPa) initially and falls to 1.8 GPa (0.8–3.7 GPa) in the long term because of mortar creep. Even so, the pier remains far from its critical load: *N*/*N_cr_* is 0.06 (0.03–0.12) initially and 0.12 (0.05–0.28) in the long term. The eccentricity amplification factor increases from 1.07 (1.03–1.14) to 1.13 (1.06–1.39) (Figure 6a, Table 8), and the resultant never leaves the core of the section. The maximum edge stress reaches 2.5 MPa (2.0–3.0 MPa); referred to the dry stone, the central safety factor is 3.1 and β*_N_* = 6.1 (Table 5). Scenario B gives practically the same amplification (1.12; 1.05–1.37), and a correlation ρ(*E_m_*, φ) = −0.8 leaves it unchanged (1.13; 1.05–1.49).

For the cantilever bound, the situation changes qualitatively. NNcr is 0.35 (0.20–0.63) initially and 0.65 (0.32–1.48) in the long term; the critical load is reached or exceeded in 20% of the simulations in scenario A (17% in scenario B), and in 49% the amplified resultant leaves the core of the section (Figure 6b, Table 8). In the cases that remain stable, the median amplification is 2.4.

The amplification factor of the braced pier depends mainly on the mortar modulus (−0.58), the joint thickness (+0.50), the effective length factor (+0.39) and the creep coefficient (+0.39).

### 3.5. H4: Buttressing of Bay 7

Since the flexural and tensile strengths of the Beauvais chalk have not been measured, H4 cannot be quantified with the same traceability as the other hypotheses. Two pieces of evidence are nevertheless relevant. First, the exterior supports of the buttressing system, unlike the choir piers, are exposed to the weather, so the saturated state governs them; the compressive strength of the M-CPI chalk drops by 42% on saturation [24], and in chalks of the Anglo-Paris Basin the loss in strength and modulus on wetting reaches 40–50%, beginning at low degrees of saturation [28]. Second, Murray documents in the building itself the failure of small units under an analogous overhanging support [10].

### 3.6. Sensitivity Synthesis

In the quantified hypotheses, the elastic modulus of the lime mortar is the parameter that most affects the results, followed by joint thickness, stone modulus and creep coefficient. Except for joint thickness, bounded by the field measurements, these are parameters for which no measured data exist for Beauvais. For H1, the result is governed instead by the stone strength, and the proxy uncertainty (scenarios A and B) changes the magnitude of the margin but not the conclusion that it is far smaller than assumed in earlier analyses. For H3, the boundary condition dominates all material parameters.

## 4. Discussion

### 4.1. Heyman’s Premise

The results do not contradict the core of Heyman’s analysis. His safe theorem [8] remains valid: if a thrust line exists within the masonry and the geometry does not change appreciably, the structure is stable, and the safety factors obtained in H1, all greater than one for the interior piers when referred to the stone, are consistent with the choir standing for twelve years. What the data do not support is the auxiliary premise that the strength of the material is irrelevant [3]. That premise rested on a comparison with a generic stone of 150–400 kg/cm^2^; with a chalk of the Beauvais type, whose strength is 7.7 MPa dry and 4.5 MPa saturated [24], the central safety factor at the base of the piers is 4.3 dry and 2.5 saturated, and even under the more favourable proxy envelope it does not exceed about 6.6. A margin of this order does not imply failure, but it means that the material is no longer negligible: any local stress concentration, eccentricity or loss of section consumes a significant part of the available reserve.

There is a second limit, already pointed out by Mainstone [3]: the theorem assumes that the geometry does not change. The spread of almost one metre measured by Goodyear between the springings of the rebuilt vault [3] and the progressive bending of the aisle piers of division 8 (Figure 1b), documented by Murray at the end of the nineteenth century [1], show that the geometry of Beauvais has changed, and that it has done so over time.

### 4.2. Viollet-le-Duc and the Objection of Wolfe and Mark

H2 provides quantitative support for the mechanism proposed by Viollet-le-Duc [2,3]. With the available data, coursed masonry tends to shorten in the long term about 4 to 6 times more than a monolithic shaft under the same stress. Where both elements work together, as in the supports between the upper and lower flying buttresses described by Viollet-le-Duc (Figure 2d), compatibility cannot be maintained without redistribution: the load migrates from the masonry to the shaft as the mortar deforms and creeps, and under full compatibility the shaft carries 2.5 to 5 times the mean stress. These factors are comparable to the safety factors of the stone itself, so the mechanism is capable of bringing a shaft close to its strength without any increase in external load. The result does not depend on the mortar being weak, but on its being far more deformable than the stone and on its slow hardening [29,31]. It also depends strongly on how stiff the mortar actually was: with a modulus of 1 GPa or more the effect becomes moderate (Figure 5b). The mechanism is therefore compatible with the data, not demonstrated by them, and its confirmation requires the measurement of the original mortar.

The refutation attributed to Wolfe and Mark [1,9] does not invalidate this result, because it was obtained with a model that could not contain the mechanism: a continuous, elastic and homogeneous photoelastic material has no joints, does not shrink and does not creep. Wolfe and Mark’s own results, which pointed to the weakness at the base of the intermediate uprights [9], are in fact compatible with those obtained here: both locate the critical point in the buttressing system rather than in the large choir piers.

### 4.3. Mainstone, Como and Time-Dependent Deformation

Mainstone’s objection [3] and Como’s conjecture [12] share a common basis: the time-dependent deformation of the masonry. The results confirm that basis and show that its consequences depend on the lateral restraint of the pier. In the main choir piers, mortar creep roughly halves the effective modulus of the masonry. If the pier is effectively braced at the level of the capitals, NNcr remains low and the eccentricity is amplified by only about 13%; within the limits of this screening model, delayed buckling of a braced pier does not emerge as a likely mechanism. If the lateral restraint is weak, the same creep brings the pier close to or beyond its critical load in a significant fraction of the simulations, and delayed instability cannot be excluded. This conditional result links H3 with Murray’s explanation: the deficiency of the buttressing that Murray identifies in bay 7 [1,10] is also a deficiency of the lateral restraint of the supports, and its effect would be aggravated by the time-dependent loss of stiffness of the masonry. Time-dependent deformation is also relevant in the slender and composite elements of the system—intermediate uprights, en délit shafts and piers of reduced mass—which is precisely where Murray documents the progressive bending observed in the nineteenth century [1]. The upper supports between the flying buttresses, to which Mainstone’s remarks also refer, have not been modelled here, because their geometry and loads are not documented with sufficient precision.

### 4.4. Murray, Weathering and Bay 7

Murray’s explanation, which locates the origin of the collapse in the insufficient buttressing of bay 7 [1,10], is geometric in nature, but its functioning depends on the material. The outer buttress overhanging the outer aisle only works if the units at its base resist bending [10], and these units, unlike the choir piers, are exposed to rain (Figure 1a,c). The M-CPI chalk loses 42% of its compressive strength on saturation [24], and chalks of the same basin lose 40–50% of their strength and modulus on wetting [28]. A comparable or greater loss in tensile strength, which governs corbel action, is to be expected. The combination of an overhang, a soft stone and permanent exposure to moisture turns the geometric weakness identified by Murray into a material weakness.

### 4.5. Amiens as a Control Case

The comparison with Amiens makes it possible to isolate the role of geometry. Both cathedrals used a chalk of the same geological age [1,18]. According to Murray, the builders of Beauvais also adopted a pier type identical to that of Amiens and placed the triforium sill at the same height, about 21 m [1]. The difference lies above: around the middle of the thirteenth century a new plan was introduced at Beauvais that raised the superstructure some five metres above the original design, together with the addition of intermediate uprights to the buttressing [1]. With the initial plan, the vault would have stood at about 41 m, close to Chartres and Bourges [1]. Beauvais and Amiens thus share material and lower elevation; they differ in a taller superstructure carried by a more slender and complex buttressing system. This observation is consistent with the results: the material is not enough to explain the collapse, but it reduced the margin with which the building could absorb that increase in height. Reims, without published mechanical data on the Courville stone, remains a lithological contrast—a Lutetian limestone of highly variable porosity [19]—and a line for future work.

### 4.6. Implications for Conservation and Strengthening

The mechanisms examined here are not only of historical interest; they bear directly on how buildings of this type should be assessed and, where necessary, strengthened. The international principles for the structural restoration of architectural heritage require that interventions be based on an understanding of the causes of damage, be compatible with the original materials, and be as limited and, where possible, as reversible as the safety of the structure allows [43]. The results suggest four implications.

First, the deformability of the joints is a structural parameter, not only a matter of durability. Repointing or grouting with mortars much stiffer than the original air lime would reduce the differential shortening of H2, but at the cost of creating stiff joints in a soft, porous stone, with the known risks of moisture trapping, salt damage and concentration of stresses in the stone; lime-based repair mortars with mechanical and hygric properties close to those of the substrate remain the appropriate choice [30]. Second, the sensitivity of the chalk to water makes moisture control—roofs, gutters, drainage of the flyers and of the buttress offsets—a structural measure for the exposed elements of the buttressing system (H4).

Third, where slender supports or monolithic shafts need additional capacity or ductility, confinement with inorganic-matrix composites is the strengthening technique most consistent with these principles. FRCM and TRM systems use a mortar matrix that is more compatible with masonry and more permeable to vapour than the epoxy of fibre-reinforced polymers [20]. Recent tests on rubble stone columns confined with stainless-steel FRCM jackets showed increases in peak load of up to about 27% and more than a doubling of ductility, with the masonry core governing the ultimate capacity, and a confinement model that accounts for stone–mortar friction predicted the confined strength within about ±10% [21]. In a building such as Beauvais, any such jacketing would have to be weighed against its visual impact on exposed ashlar and limited to elements where the deficit is demonstrated; the gain in ductility is especially relevant for supports, like those of H2 and H3, whose vulnerability arises from redistribution and loss in stiffness rather than from lack of mean strength.

Fourth, the sensitivity analysis provides a rational order for diagnostic investigation before any intervention: sampling of the mortar to determine its degree of carbonation and stiffness, minor-destructive tests of the stone (dry and saturated strength and modulus, tensile strength), and monitoring of the deformation of the slender supports. The probabilistic framework used here can then be updated with those data to assess the existing margin with less uncertainty.

### 4.7. Limitations

The study has several limitations. The tested chalk sample is a geological proxy from a quarry in the same formation, but not from the main quarry of the works [1,24]; scenario B bounds this uncertainty but cannot replace tests on the cathedral stone. The mortar values correspond to modern, young air lime mortars and not to the original mortar, which has not been characterised. The stone modulus is estimated from its strength, not from a test. Most load components are estimates from volumes, anchored on the single documented resultant of Benouville. The masonry measurements are limited to the lower zone, on a weathered exterior face and with a reading step of 1 cm, comparable to the joint thickness, and the upper-zone ranges are assumptions. The inputs are assumed independent except in the correlation cases examined. EN 1996-1-1 is used outside its calibration domain, and only as a lower bound. The models are screening models: H2 is bracketed by two limiting states, H3 replaces the compound pier by an equivalent homogeneous column with Euler amplification, and neither represents cracking, non-linear behaviour or the three-dimensional interaction of the structure. These limitations have been addressed explicitly through ranges and sensitivity analysis, which show that the qualitative conclusions on H1 and H2 are stable over the interval considered, whereas the conclusion on H3 depends on the lateral restraint of the pier.

## 5. Conclusions

This study answers the question in its title with a qualification: the material properties do not explain the 1284 collapse on their own, but the results are compatible with a material contribution to it. Specifically:

1. The central safety factor against crushing of the choir piers, computed with the measured strength of a chalk representative of the Beauvais stone, is 4.3 dry and 2.5 saturated (up to 6.6 and 3.9 if the cathedral stone were stronger than the proxy). It does not explain the collapse, but it invalidates the premise that the material was irrelevant.
2. The difference in deformability between masonry with lime joints and monolithic shafts is large and grows over time. Under strain compatibility it concentrates in the shafts stresses of 2.5 to 5 times the mean value, comparable to the margin of the stone. The mechanism proposed by Viollet-le-Duc is compatible with the data, and the refutation based on photoelastic models does not reach it.
3. Mortar creep halves the long-term stiffness of the masonry. In a pier that is effectively braced it produces only a small amplification of the eccentricity; if lateral restraint is lost, delayed instability becomes plausible. The vulnerability of the piers to creep is therefore conditional on the buttressing, which connects the hypotheses of Como and Murray.
4. The sensitivity of the chalk to water weakens precisely the exterior elements where Murray locates the origin of the collapse, which allows his geometric explanation to be read in material terms as well.
5. The parameter on which the results depend most, the modulus of the original mortar, has never been measured. Its characterisation is the condition for settling the question.

These conclusions are subject to the limitations of the data and models. The stone was characterised through a proxy, the mortar through modern analogues, the upper-zone masonry and the creep coefficient through working assumptions, and the mechanisms through screening models that do not represent cracking or the global behaviour of the structure. Future work should address, in order of priority: (i) the characterisation of the original mortar of the choir—degree of carbonation inside the piers, elastic modulus and creep; (ii) the modulus and the compressive and tensile strengths of the cathedral chalk, dry and saturated, from samples of the fabric or of the carrière Saint-Pierre; (iii) a survey of joint geometry on thirteenth-century interior faces and in the upper zone with sub-millimetre resolution; (iv) the use of the plumb-line data in Sauvageot’s restoration drawing, cited by Murray [1], to quantify the time-dependent deformation of the piers of division 8; and (v) a numerical model of a complete bay, with non-linear and time-dependent material laws calibrated on those data, to verify the screening results and to evaluate compatible strengthening strategies.

## Figures and Tables

**Table 1:** Properties of the Craie de Picardie, sample M-CPI [24].

Property	Value (±as Reported)	Test Standard
Uniaxial compressive strength, dry (*R_c_*)	7.7 ± 0.8 MPa	NF B 10-509
Uniaxial compressive strength after 48 h immersion (*R_c_*_48_)	4.5 ± 0.3 MPa	NF B 10-509
Total porosity	34.9 ± 0.8%	NF B 10-503
Porosity accessible to water at 48 h	26.3 ± 0.8%	NF B 10-503/504
Water absorption coefficient *S*_48_	0.75 ± 0.01	NF B 10-504
Mean pore access radius	0.43 µm	Mercury porosimetry
Dry bulk density (estimated)	≈1.76 g/cm^3^	From porosity

**Table 2:** Components of the axial load at the base of a choir pier (t; 10^5^ simulations).

Component	Model and Distribution	Source	P50 (P5–P95)
Resultant at the tas-de-charge, *N_cc_*	U(105, 111)	[3,7]	108 (105–111)
Upper pier and wall strips, 40.25–21 m	(40.25 − 21)·*A_up_*·ρ; *A_up_*~*U*(1.0, 1.8) m^2^	[1]; estimate	51 (37–64)
Tracery, glazing, triforium passage	*U*(10, 25)	Estimate	18 (11–24)
Arcade spandrels	1.34·[8.5·(21 − *L*) − *A_open_*]·ρ	[1]; estimate	68 (57–79)
Inner aisle vaults and roof (share)	*U*(10, 25)	Estimate	17 (11–24)
Pier self-weight	*A*·*L*·ρ	[1]; estimate	48 (43–53)
Total axial load *N*	Sum		309 (288–331)
Mean stress σ = *N*/*A* (MPa)	*A*~*U*(1.60, 1.80) m^2^	[1]	1.78 (1.64–1.94)

ρ~*U*(1.76, 2.00) t/m^3^; *L*~*U*(14, 16) m; *A_open_* = *c*·7.1*·f*, with *c*~*U*(0.65, 0.75) and arch rise *f* = 21 − *L* − *U*(0.8, 1.5) m.

**Table 3:** Input variables of the Monte Carlo models.

Variable	Symbol (Unit)	Distribution	Source/Status	Used In
Stone strength, dry	*R_c_* (MPa)	A: N(7.7, 0.8); B: LU(6.2, 20)	[24,27]	H1–H3
Stone strength, saturated	*R_c48_* (MPa)	A: N(4.5, 0.3); B: *R_c_*·N(0.584, 0.05)	[24]	H1
Modulus ratio	*MR* = *E_s_*/*R_c_* (–)	U(500, 1500)	[40,41]	H2, H3
Mortar modulus	*E_m_* (MPa)	LU(125, 500); sens. 1000, 2000	[29]; assumption	H2, H3
Mortar creep coefficient	φ (–)	U(0.5, 3.0)	Assumption; [35]	H2, H3
Mortar strength	*f_m_* (MPa)	1.23 (fixed)	[29]	H1
Shape factor EN 772-1	δ (–)	U(0.80, 0.95)	[38]; assumption	H1
Joint thickness, lower zone	*t* (mm)	U(5, 20); sens. U(5, 25)	Author’s survey	H2, H3
Course height, lower zone	*h* (mm)	U(280, 320)	Author’s survey	H2, H3
Joint thickness, upper zone	*t* (mm)	U(15, 30)	Assumption [1,7]	H2
Course height, upper zone	*h* (mm)	U(250, 400)	Assumption [1,7]	H2
Masonry density	ρ (t/m^3^)	U(1.76, 2.00)	[24]; estimate	H1, H3
Load components	(t)	Table 2	Table 2	H1, H3
Resisting area of the pier	A (m^2^)	U(1.60, 1.80)	[1]	H1, H3
Free height	*L* (m)	U(14, 16)	[1]; estimate	H1, H3
Initial eccentricity	*e*_0_ (m)	U(0.02, 0.10)	Assumption	H3
Effective length factor	*K* (–)	Braced: U(0.7, 1.0); cantilever: 2	Assumption	H3
Shaft area fraction	*a* (–)	0.05, 0.10, 0.20 (cases)	Assumption	H2

N: normal (mean, SD); U: uniform (min, max); LU: log-uniform (min, max).

**Table 4:** Ratio between resistance and mean stress at the base of the choir piers (5th, 50th and 95th percentiles) for the two stone scenarios.

Resistance Considered	A: P5	A: P50	A: P95	B: P5	B: P50	B: P95
Dry stone (*R_c_*)	3.51	4.31	5.16	3.67	6.25	10.61
Saturated stone (*R_c_*_48_)	2.19	2.52	2.88	2.09	3.64	6.32
Masonry, EN 1996-1-1, mean (1.2 *f_k_*)	0.89	1.04	1.22	0.92	1.36	1.98

**Table 5:** Central and characteristic safety factors, reliability indices and probability that the resistance is exceeded at the base of the choir piers (H1) and at the edge of the braced pier in the long term (H3).

Check	Scenario	γc=μRμS	γk=R5%S95%	β*_N_*	β*_LN_*	*P_f_* (MC)
H1, dry stone	A	4.31	3.29	7.34	12.6	<10^−5^
H1, saturated stone	A	2.52	2.06	8.63	11.0	<10^−5^
H1, masonry EN 1996-1-1	A	1.04	0.83	0.44	0.45	0.33
H1, dry stone	B	6.60	3.39	2.54	5.58	<10^−5^
H1, saturated stone	B	3.86	1.94	2.14	3.86	<10^−5^
H1, masonry EN 1996-1-1	B	1.39	0.86	1.17	1.36	0.13
H3 braced, σ_max_ vs dry stone	A	3.12	2.14	6.10	7.02	<10^−5^
H3 braced, σ_max_ vs dry stone	B	4.79	2.21	2.36	4.40	<10^−5^

Reference value of EN 1990 for a 50-year period: β = 3.8 [42] (for comparison only). *P_f_* < 10^−5^: no failure in 10^5^ simulations (β*_MC_* > 4.3).

**Table 6:** H2: ratio *R* between the shortening of coursed masonry and that of a monolithic shaft (equal-stress limit) and stress concentration in the shaft (equal-strain limit), scenario A; median (5th–95th percentile).

Result	Lower Zone (Measured)	Upper Zone (Assumed)
*R*, instantaneous	2.07 (1.35–4.00)	2.81 (1.67–5.60)
*R*, long term	3.91 (1.88–9.78)	5.94 (2.69–14.69)
Share of simulations with long-term *R* > 2	93%	99%
σshaftσ¯, long term, *a* = 0.05	3.41 (1.80–6.80)	4.76 (2.48–8.72)
σshaftσ¯, long term, *a* = 0.10	3.03 (1.72–5.21)	3.98 (2.30–6.20)
σshaftσ¯, long term, *a* = 0.20	2.47 (1.60–3.55)	2.99 (2.01–3.93)

**Table 7:** H2: sensitivity of the long-term ratio *R* to the main assumptions; 5th/50th/95th percentiles.

Case	Lower Zone	Upper Zone
Baseline (scenario A, independent inputs)	1.88/3.91/9.78	2.69/5.94/14.69
Stone scenario B (proxy envelope)	2.14/5.24/15.53	3.17/8.21/23.85
Lower-zone joints 5–25 mm (quantisation)	1.97/4.41/11.64	–
ρ(*E_m_*, φ) = −0.5	1.77/3.91/10.89	2.47/5.97/16.37
ρ(*E_m_*, φ) = −0.8	1.72/3.91/11.52	2.35/6.00/17.32
ρ(*R_c_*, *MR*) = −0.5	1.91/3.91/9.49	2.76/5.93/14.16
*E_m_* up to 1000 MPa	1.49/3.03/8.77	1.93/4.46/13.22
*E_m_* up to 2000 MPa	1.26/2.41/8.05	1.49/3.41/12.16

**Table 8:** H3: effective modulus, ratio between axial and critical load and amplification factor of the choir pier (scenario A); median (5th–95th percentile).

Result	Braced, Initial	Braced, Long Term	Cantilever, Initial	Cantilever, Long Term
*E_f_* (GPa)	3.39 (1.97–5.80)	1.84 (0.83–3.74)	3.39 (1.97–5.80)	1.84 (0.83–3.74)
*N*/*N_cr_*	0.06 (0.03–0.12)	0.12 (0.05–0.28)	0.35 (0.20–0.63)	0.65 (0.32–1.48)
α (stable cases)	1.07 (1.03–1.14)	1.13 (1.06–1.39)	1.55 (1.25–2.67)	2.38 (1.43–14.9)
Share *N* ≥ *N_cr_*	0%	0%	0%	20%
Share α·*e*_0_ > *D*/8	0%	0%	6%	49%

## Data Availability

All data derive from the published sources cited or from the author’s measurements and are included in Table S1. The calculation code is provided as Code S1.

## Supplementary Materials

The following supporting information can be downloaded at: https://www.mdpi.com/article/10.3390/ma19214659/s1, Table S1: Traceable database of geometric, load and material parameters, input distributions and results, with sources; Code S1: Python script reproducing all results and Figure 4, Figure 5 and Figure 6 for hypotheses H1–H3.

## Abbreviations

*A*, area of the pier section; *a*, shaft area fraction; BRGM, Bureau de Recherches Géologiques et Minières; *D*, equivalent diameter; *e_0_*, initial eccentricity; *E_f_*, *E_m_*, *E_s_*, effective modulus of coursed masonry, modulus of mortar and of stone; FRCM, fabric-reinforced cementitious matrix; *f_b_*, normalised unit strength; *f_k_*, fmean, characteristic and mean strength of masonry; *f_m_*, mortar strength; *h*, course height; *I*, moment of inertia; *K*, effective length factor; *L*, free height; M-CPI, Craie de Picardie sample of BRGM; MR, modulus ratio; *N*, axial load; *N_cr_*, critical load; *P_f_*_,_ probability that the resistance is exceeded; *R*, free-strain ratio of masonry to shaft; *R_c_*, *R_c_*_48_, compressive strength of the dry and saturated stone; *t*, joint thickness; TRM, textile-reinforced mortar; α, amplification factor; β*_N_*, β*_LN_*, β*_MC_*, reliability indices; γ*_c_*, γ*_k_*, central and characteristic safety factors; δ, shape factor; ρ, density or correlation coefficient; σ, $\bar{\sigma }$, mean stress; σ_max_, edge stress; φ, creep coefficient of the mortar.
